# Measurement and control of mechanics of cardiac trabeculae secured by light‐curable hydrogel

**DOI:** 10.1113/EP093087

**Published:** 2025-10-29

**Authors:** Emily J. Clark Murphy, Toan Pham, June‐Chiew Han, Kenneth Tran, Khoon S. Lim, Poul M. F. Nielsen, Andrew J. Taberner

**Affiliations:** ^1^ Auckland Bioengineering Institute (ABI) University of Auckland Auckland New Zealand; ^2^ School of Medical Sciences University of Sydney Sydney Australia; ^3^ Department of Engineering Science and Biomedical Engineering, Faculty of Engineering University of Auckland Auckland New Zealand

**Keywords:** cardiac trabecula, hydrogel, image registration, real‐time

## Abstract

Isolated cardiac trabeculae are small heart muscle tissue preparations, which have been widely used in in vitro studies of mechanics and energetics function of cardiac muscle. Current instruments for such experimentation often (1) involve delicate mounting of the muscle, (2) constrain investigations to one muscle at a time, and thus (3) limit experimental throughput. Here, we present a novel device that allows trabeculae to be secured by a visible‐light photo‐crosslinked hydrogel, manipulated via a robust motor‐driven stainless steel cantilever, and their shortening and force production to be measured and controlled using feedback from real‐time imaging. The device has multiple wells, making it amenable to high‐throughput testing of muscle. We use our robust, accurate image registration techniques to measure cantilever and gel deformation during trabecula contraction and thereby provide a measure of trabecula shortening and force production during twitches. We apply methods to allow the trabecula to contract either isometrically or isotonically. The methods used in this device can be widely applied to the study of the mechanics of cardiac muscle samples in laboratories with available light microscopic systems.

## Abstract (highlights)

- **What is the central question of this study?**
  Existing devices for trabecula experimentation are challenging to use as they require manually attaching the muscle at each end to sutures/hooks/clamps/pins, and usually involve the direct manipulation, using forceps, of the delicate tissues: can a better device be constructed?
- **What is the main finding and its importance?**
  Methods were developed for constraining cardiac trabeculae with hydrogel and applying feedback‐control during contractions. These methods are applicable across many available light microscope systems, and may in future allow parallelized study of cardiac trabeculae.

## 1 INTRODUCTION

Cardiac trabeculae are multicellular heart muscle tissues that contain hundreds to thousands of axially aligned myocytes. They can be dissected intact from the inner surface of the ventricles. These tissues have been extensively used as isolated tissue preparations for studying in vitro cardiac ionics (Lamont et al., 1998; Lookin et al., 2023), mechanics (Choi et al., 2020; de Tombe & Ter Keurs, 1990; Garrett et al., 2019; Layland & Kentish, 2000; Lookin et al., 2023) and energetics (Han et al., 2023; Mellor, 2020; Pham et al., 2017). Trabeculae are sufficiently small (typically 2–3 mm in length and 100–200 µm in diameter) to permit adequate oxygenation by diffusion, even under high energy demand conditions (Han et al., 2011). However, their small size and delicate nature make them difficult to handle and susceptible to damage, thereby limiting experimental throughput.

Instruments available for isolated trabecula experimentation usually require securing the tissue at both ends to the apparatus – typically to a force sensor at one end and a length actuator at the other end. Attachment of a muscle to the sensor and actuator is typically made using hooks, clamps, sutures, pins or adhesives (Bupha‐Intr et al., 2007; Janssen et al., 1998; Pham et al., 2022; Qin et al., 2020). However, operator‐induced tissue damage can easily result during the mounting process when manually manipulating the delicate tissue to the attachment points. The process requires a great deal of skill and dexterity in tissue handling and can be very time‐consuming.

A common method for measuring force production of these delicate tissue preparations is to use piezoresistive silicon strain gauges (de Tombe & Ter Keurs, 1990; Lamont et al., 1998; Layland & Kentish, 2002; Power et al., 2018). While this method is simple and effective, these force sensors are fragile, prone to thermal drift and frequently break due to accidental damage during experiments. A more robust alternative method is to measure the deflection of a stainless steel cantilever using a heterodyne or Fabry–Pérot laser interferometer. The surface of the cantilever provides sufficient reflection to measure cantilever deflection, and the corresponding force production can be measured with high precision (Tabern et al., 2018). However, the complexity of the laser interferometer system makes it difficult to implement, particularly in laboratories where more simple set‐ups may be preferable.

Recently, hydrogels have gained significant interest in tissue engineering applications for creating artificial tissues, implantable therapies, enhancing cell protection (Asl et al., 2024; Hinderer et al., 2015; Lim et al., 2015), and cardiac repair and regeneration (Li et al., 2023). Hydrogels are suitable for use in cell manipulation and tissue handling applications due to their ability to absorb water, promote nutrient diffusion, bind to integrins in the cell membrane, and be manufactured with a range of mechanical properties (Sun et al., 2018). The design of polymer backbones and the incorporation of peptides or proteins allow hydrogels to closely mimic the extracellular matrix structure and composition (Lim et al., 2019). Embedding cells in such hydrogels permits non‐contact handling and avoids stress concentration at the cell membranes, hence minimising tissue damage that might be caused by human manipulation. In addition, hydrogels with known mechanical properties can potentially serve as force sensors to estimate the force production of embedded cardiac muscle specimens (Lam Po Tang et al., 2023) from measurement of the deformation of the surrounding gel. These advantages provide the impetus to extend the application of hydrogels in mounting and securing isolated contracting cardiac muscle in vitro.

We recently exploited light‐curable hydrogels (Lim et al., 2019) to secure cardiac trabeculae into a microscope‐slide‐based instrument, thus minimising the limitations associated with manual tissue mounting (Lam Po Tang et al., 2023). Our instrument secures one end of a trabecula to the base of a microscope‐slide‐sized eight‐well plate using light‐curable hydrogel. The deformation of the hydrogel can be tracked to infer trabecula force production. The other end of the trabecula is secured to a stainless steel cantilever force sensor attached to a micromanipulator. This approach provides more efficient mounting of a specimen with little manual contact.

In this study, we expand upon this approach to track muscle force production and length change in real‐time from camera images. In addition, we use our image processing techniques to measure sarcomere length in real‐time, allowing the muscle to be stretched to a precisely known operating length before experiments. We incorporate into the device a high‐stroke piezoelectric motor to dynamically control muscle length during each contraction. Image‐based feedback control of muscle length or force permits us to develop different contraction modes: isometric and isotonic contraction, respectively. We demonstrate the use of this system in experiments to determine and control muscle force, muscle length, and gel displacement in real‐time during experiments on cardiac trabeculae.

## 2 METHODS

### 2.1 Ethical approval

The animal handling procedures were conducted in accordance with protocols approved by the Animal Ethics Committee of The University of Auckland (AEC 22653). A male Wistar rat was used in this study. The rat was provided by the Vernon Jansen Unit, the laboratory animal facility of the University of Auckland. It was housed in a 12/12 h light–dark cycle at room temperature (22°C) with ad libitum access to standard rat chow and water. The rat was brought to our laboratory (Auckland Bioengineering Institute within the University of Auckland) and placed in a climate‐controlled chamber with access to food and water for at least 1 h to minimise any stress arising from the transportation.

### 2.2 Device

**FIGURE 1:**
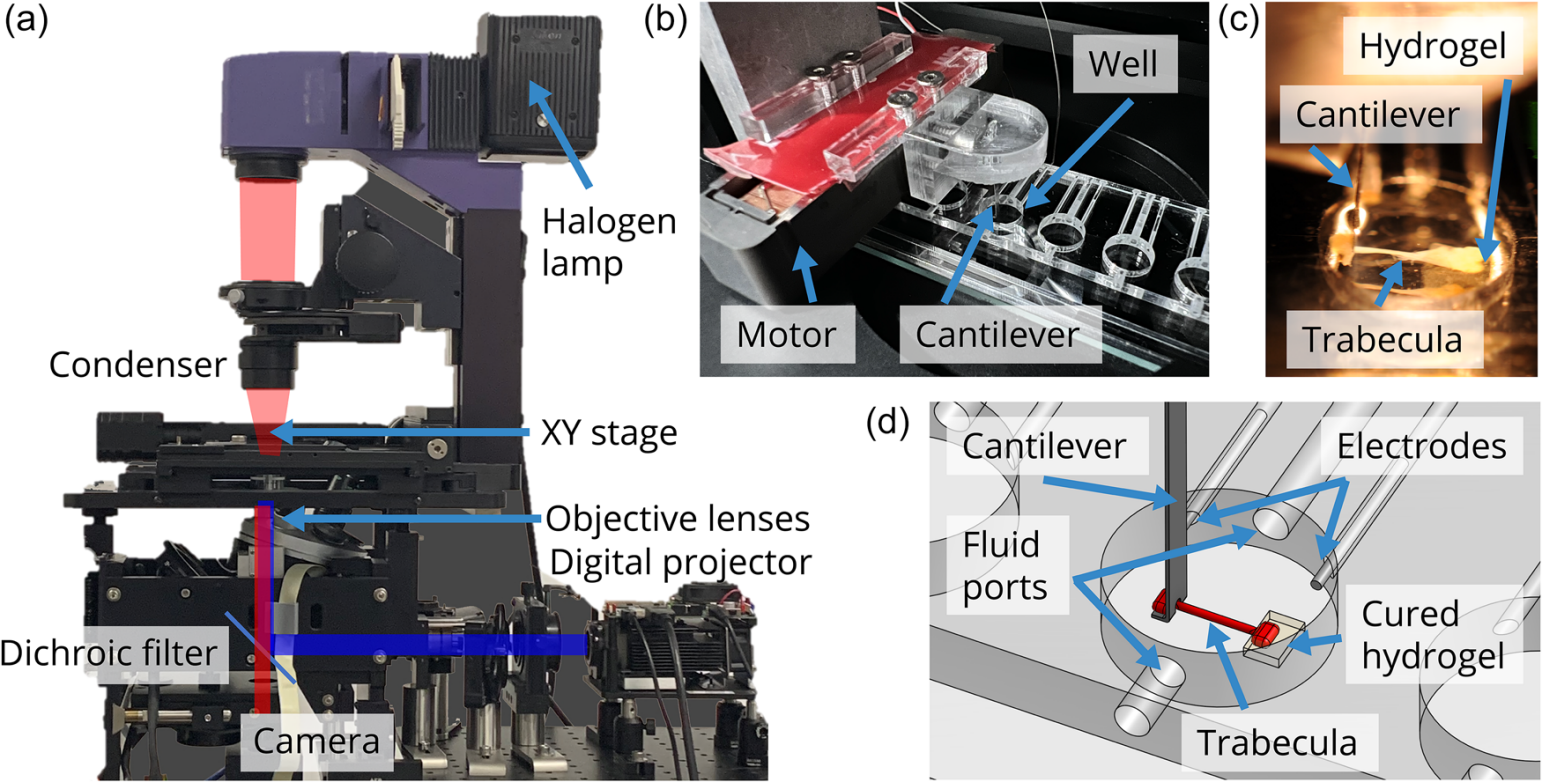
(a) Imaging and printing system, with imaging (red) and projection‐printing (blue) light paths. (b) Cantilever manipulator and actuator. (c) Photograph of a trabecula in a well (6 mm diameter), secured by the cantilever (left) and hydrogel (right). The trabecula was approximately 270 µm in diameter and 1760 µm in length. (d) Detailed diagram of a single well with trabecula.

Our instrument is developed from a modified inverted microscope (Discovery I, Molecular Devices, San Jose, CA, USA) comprising a transmission imaging system (Nikon, Tokyo, Japan), 6‐lens motorized turret with lens focusing system, and motorized *X–Y* positioning stage (Prior, Rockland, MA, USA). To this, we have added a custom microscope slide assembly containing eight experimental wells, 3D positioning stage, piezo‐electric motor with attached custom cantilever, digital light projection system and a custom user interface (Figure 1). The device is rendered light‐proof by shrouding with black‐out curtains on a vibration‐free optical table. The motorized *X–Y* stage provides 1 µm spatial resolution and allows a well to be positioned in the field of view of the microscope.

### 2.3 Experimental slide

A line of eight wells (6 mm diameter × 2 mm height, spaced 9 mm apart) was machined from an acrylic sheet and glued to a standard glass microscope slide; data from only one well are presented here. Two platinum stimulation electrodes were placed in the well. Superfusate can be introduced via a fluid port and removed by a suction system on the opposite side. A trabecula was placed in the well with solution and/or gel, and imaged, manipulated, or light‐cured in place with hydrogel. The assembly was positioned in the view of the microscope imaging system using the *X–Y* stage.

### 2.4 Imaging system

The trabecula was imaged in brightfield mode with red (>600 nm) illumination, to avoid premature curing of the hydrogel, which cures when exposed to blue light (400–480 nm). Light that passed through the specimen was sampled through a ×2 objective lens (Nikon Plan APO, 0.1 numerical aperture (NA)) during mounting and positioning. A 20× objective lens (Nikon LWD, 0.4 NA) was used to image sarcomeres. A machine‐vision camera (FLIR USB3 Blackfly S, BFS‐U3‐23S3M‐C, 1920 pixel × 1200 pixel, Wilsonville, OR, USA) captured full‐frame video recordings of the trabecula at a rate of up to 164 frames per second. The spatial resolution of the imaging system (with ×2 lens) was determined with a diffraction grating to be 1.71 µm/pixel.

### 2.5 Projector

To pattern and cure the hydrogel, a LED projector (TI DLP4710EVM‐LC, 1920 pixel × 1080 pixel, Dallas, TX, USA) was attached to the microscope's rear port and aligned with the camera system. In this apparatus, we used a visible‐light Type II photo‐initiating system (Tris‐bipyridyl‐ruthenium (II) hexahydrate (Ru) and sodium persulfate (SPS)), which cross‐links the hydrogel when excited by 400–480 nm light. The blue (450 nm) LED on the projector was used to project light patterns onto the gel. The user controlled the brightness and exposure time of the projected image using the computer program described below in the section ‘Software’.

### 2.6 Force sensor

A force sensor cantilever (11.25 mm long, 0.96 mm wide) was manufactured from 100 µm‐thick stainless steel shim. Two prongs (<500 µm long) protruding from the cantilever tip were bent 90° to form a pair of hooks, that can hold one end of the trabecula in place. The cantilever was fixed to a piezoelectric motor (APF705, Thorlabs, Newton, NJ, USA), which was attached to a manually adjustable *X–Y*–*Z* stage (M‐DS25‐XYZ, Newport, Irvine, CA, USA). The linear stages were fixed to the *X–Y* stage of the microscope. This allowed the cantilever to be controlled manually to secure, lift and position one end of the muscle, without requiring forceps. This also allowed muscle length to be precisely and dynamically controlled with the motor using a software PID algorithm.

### 2.7 Software

A custom user interface was developed in LabVIEW 2021 (NI LabVIEW version 21.0.1f2, Austin, TX, USA, running on a Windows 11 CPU, i9‐12900F). The microscope image was displayed in real‐time, and the objectives, microscope stage and focus were controlled electronically through the software, or by joystick. Sarcomere length was reported in real‐time when using a ×20 objective lens, allowing the muscle to be stretched to a desired sarcomere length.

The software enables the user to draw multiple polygonal regions of interest on the displayed image in real‐time, and thus define sections to be photocured. The user can adjust the dimensions of each region, and the intensity at which blue light should be projected. Once designed, the patterns are projected onto the gel through the objective lens for a user‐defined period. Here, we used the hydrogel to attach one end of the muscle to a glass slide and manipulated the other end using the cantilever.

Once in place, the trabecula was electrically field‐stimulated by two platinum electrodes. Frequency, duration and amplitude of the electrical stimulus can be controlled. Stimulus data and muscle images were recorded throughout the experiment. The images were saved to raw binary files and stimulus data to a text file.

The user can choose areas/points on the live image to track in real‐time. The user can also choose to control muscle length or force via the cantilever using any combination of the tracked deformations. Feedback control of the selected variable (length, force) was effected by a software PID algorithm driving the piezoelectric motor via an amplifier (LVPZT, Physik Instrumente, Karlsruhe, Germany).

### 2.8 Image registration and tracking

A form of digital image correlation (DIC) was used to calculate the displacements of the cantilever, muscle and gel. Frame‐to‐frame displacements were calculated using the ‘P‐SG‐GC’ subpixel image registration algorithm developed by HajiRassouliha et al. (2017). In this study, 1D and 2D versions of the algorithm were implemented in LabVIEW, for real‐time tracking.

The 1D version of the algorithm was used to track the edge of the cantilever in real time. The user was able to draw a vertical line on the live image and the line was taken as the centre of the window in the *x*‐axis (horizontal direction) that spanned the edge of the cantilever. The edge of the cantilever was tracked in all rows using a 512‐pixel window; the edge displacements tracked from each row were averaged to provide an estimate of the cantilever displacement.

The 2D version of the algorithm was used to track features in the muscle and/or gel. The user could select up to three control points in the live image, each of which would define the centre of a 64 pixel × 64 pixel sub‐image. The user was able to choose when to start performing the calculations, at which point a reference image surrounding each point was stored. On subsequent frames, cross‐correlation was performed, comparing the moving sub‐image to the stored reference sub‐image. If either the horizontal (*x*) or the vertical (*y*) displacement between the moving and reference sub‐image exceeded 15 pixels, and the integer error was less than the threshold error (HajiRassouliha et al., 2017), then the position of the control point on the moving image was updated by adding the estimated integer displacements to the current moving image control point, to prevent the feature being tracked moving outside of the sub‐image of interest. The 1D cantilever and 2D displacements were computed at a rate of 164 Hz, presented to the PID algorithm, displayed as traces to the user, and recorded in a text file.

### 2.9 Force measurement

During fixed‐end contraction (i.e. no feedback control of muscle length), muscle force was estimated from the cantilever displacement measurement. The cantilever was tracked, as described above, using the 1D image registration algorithm. Cantilever displacement was converted into a force (*F*) using the following equation and the known properties of the cantilever:

(1) $$F=\frac{\delta Ewh^{3}}{4l^{3}}$$

where δ is the deflection of the cantilever, E is Young's modulus for stainless steel, and w,h and l are the width, thickness and length of the cantilever, respectively (Table 1).

**TABLE 1:** Material and geometrical properties of cantilever force sensor.

Property	Cantilever
E	200 GPa
w	0.96 mm
h	100 µm
l	11.25 mm

The cantilever displacement can be used to estimate force only during fixed‐end contractions when the attached piezoelectric motor holds the other end of the cantilever stationary. During piezoelectric motor motion, the tracked movement of the cantilever tip is no longer representative of the force produced. Thus, during feedback‐control protocols, the displacement of a user‐selected control point in the gel is used to estimate force. To enable this measurement, gel stiffness was calibrated by tracking gel displacement as a function of the force measured using the tracked cantilever displacement and Equation (1). The muscle was electrically stimulated at 1 Hz (6 ms, 5 V). Cantilever and gel displacement were tracked over 5 s. The cantilever displacement was converted into a force and a linear regression was performed on the force and gel displacement data using least squares fitting. The offset was set to zero, as no displacement is expected when the muscle is not producing force.

### 2.10 Real‐time length measurement

Sarcomere length was measured in real‐time using a series of 1D fast Fourier transforms (FFT) (Cheuk et al., 2020). A rectangular area on the image (at ×20 magnification) was chosen where the sarcomeres were distinct, and an FFT was performed on each row inside this region. The resulting spectra were averaged. An exponential function was then fitted to the average spectrum; frequency bins corresponding to the expected sarcomere length range were omitted (the minimum sarcomere length expected is 1.6 µm and the maximum is 2.5 µm) from this fit. The fitted exponential was subtracted from the average spectrum across all bins, and a Gaussian function was fitted to the data corresponding to the expected sarcomere length range. The centre of the Gaussian peak was then found as an estimate of the mean sarcomere length. The reader is referred to Cheuk et al. (2020) for details and results of implementing this method.

Muscle length was measured between user‐defined points on the image. Muscle length can be defined either between a point at the left end of the muscle and the cantilever on the right‐hand side, or between user‐defined points at the left end of the muscle and another point on the muscle.

### 2.11 Experimental methods

#### 2.11.1 Sample preparation

The rat (300 g) was deeply anaesthetised with isoflurane (5% in O_2_), injected with heparin (1000 IU/kg), cervically dislocated, and the heart rapidly excised. The aorta was immediately cannulated for Langendorff perfusion with 100% oxygenated, low‐Ca^2+^, Tyrode's solution (containing 130 mmol/L NaCl, 6 mmol/L KCl, 1 mmol/L MgCl_2_, 0.5 mmol/L NaH_2_PO_4_, 0.3 mmol/L CaCl_2_, 10 mmol/L HEPES, 10 mmol/L glucose), supplemented with 20 mmol/L 2,3‐butanedione monoxime (BDM) (pH adjusted to 7.4 using Tris) at room temperature. Trabeculae were excised from the endocardial surface of the right ventricle.

**FIGURE 2:**
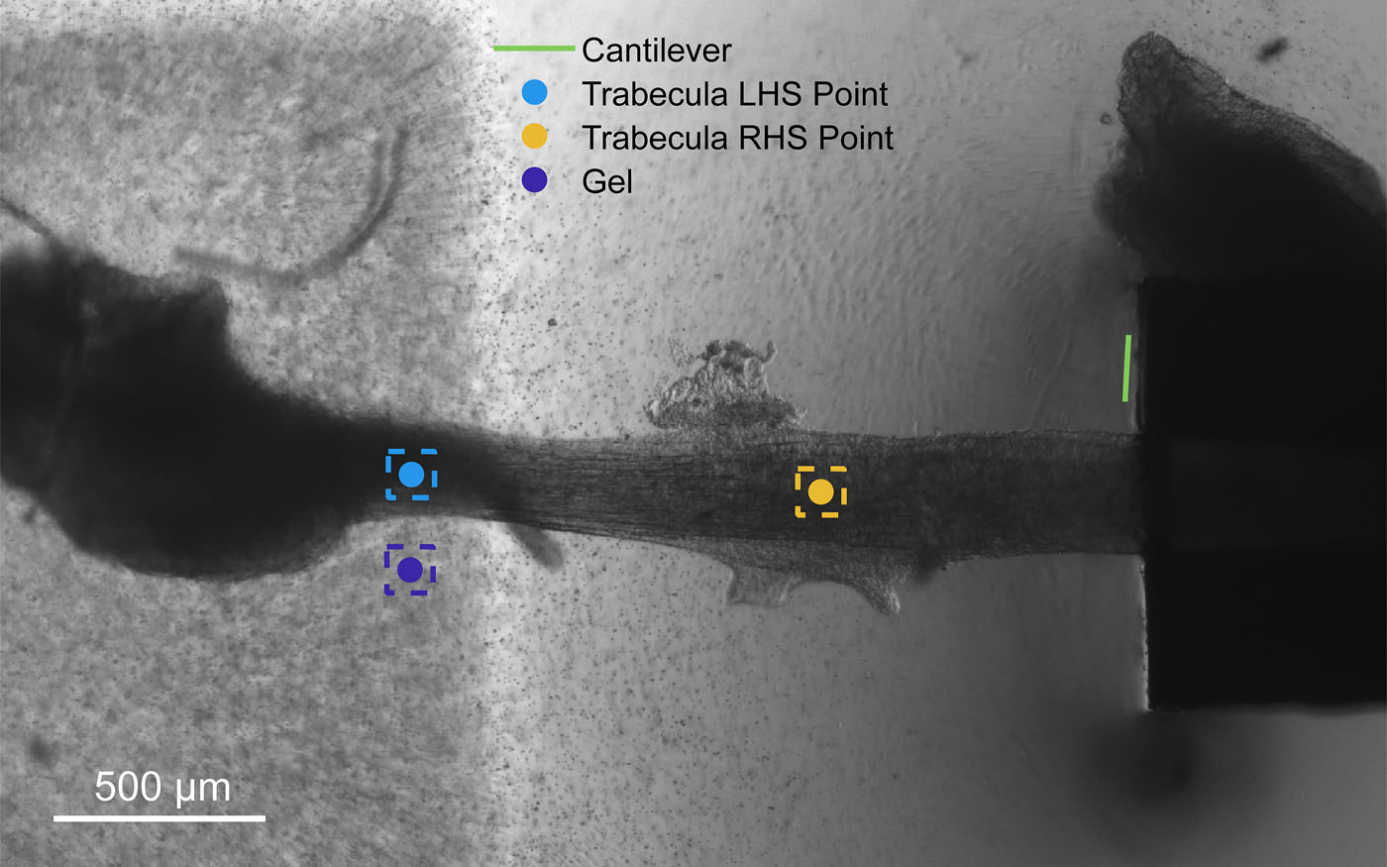
Trabecula showing areas/points tracked. Points tracked in 2D are shown, with dashed lines indicating the subimage used for tracking. Green line is where cantilever was tracked, light blue is a point at the left‐hand side of the muscle tracked in 2D; orange is a point at the right‐hand side of the muscle tracked in 2D, and the dark blue is a point in the gel tracked in 2D. The trabecula was 270 µm in diameter and 1760 µm in length.

The selected trabecula was 270 µm in diameter and 1760 µm in length (Figure 1c). Length measurements were taken between the left endpoint (light blue point) of the muscle and the edge of the cantilever (green line), shown in Figure 2.

#### 2.11.2 Hydrogel preparation

Gelatin methacrylol (GelMA) was dissolved in 1.5 mmol/L Ca^2+^ Tyrode's solution at 17.5 % w/v and kept at 37°C using a dry bath. Ruthenium (Ru) and sodium persulfate (SPS; Na_2_S_2_O_8_) were dissolved in deionised water to concentrations of 50 mmol/L and 500 mmol/L, respectively. Polystyrene beads (3.25 µm diameter) were mixed into the solution to provide visible features for gel deformation tracking.

#### 2.11.3 Experimental protocol

Experiments were performed at room temperature. The fabrication of GelMA composite gels has been described previously (Chalard et al., 2024). Briefly, 50 µL of hydrogel‐precursor solution was gently pipetted with 2 µL of beads. Ru and SPS were added to a final concentration of 1 mmol/L and 10 mmol/L, respectively. A volume of 35 µL of this mixture was added to a well. The trabecula was transferred into the well and gently submerged in the hydrogel‐precursor solution.

One end of the trabecula was fixed to the microscope slide by curing a rectangular section of hydrogel over the end tissue for 10 s at maximum brightness. Once curing was complete, the bath was flushed with fresh Tyrode's solution to remove uncured hydrogel‐precursor, and the bath remained filled with Tyrode's solution. The dimensions of the cured gel were estimated to be 1265 µm × 2000 µm × 700 µm (w×l ×h). The other end of the muscle was captured and stretched by the cantilever, while sarcomere length was measured using the ×20 objective lens. As the cantilever could be controlled with linear *X–Y* stages, attaching the other end of the trabecula and stretching the muscle could be achieved within a minute. The trabecula sarcomere length was stretched to 2.2 µm to maximise its active force production.

The trabecula was subjected to four contraction protocols.

#### 2.11.4 Protocol 1: Fixed‐end contraction

The muscle was stimulated at 1 Hz (pulse: 1333 V m^−1^, 6 ms duration) to produce a fixed‐end contraction, where muscle length was constrained only by the stiffness of the gel and cantilever. The muscle shortened slightly during this contraction as the gel and cantilever were displaced by the muscle force. Images were recorded at 164 fps. Real‐time tracking was performed on four areas on the image: the cantilever edge tracked in 1D, the lefthand side (LHS) of the muscle tracked in 2D, the righthand side (RHS) of the muscle tracked in 2D, and an area selected in the gel tracked in 2D, as shown in Figure 2. Next, the effective stiffness of the gel in this region was found by linear regression of gel displacement against the force measured by the cantilever during fixed‐end contractions, thenceforth allowing the gel to be used as a force sensor during feedback control of cantilever position for muscle length control. Linearity between gel displacement and force has previously been established in Lam Po Tang et al. (2023).

#### 2.11.5 Protocol 2: Isometric contraction protocol based on trabecula muscle length defined between end tissue and cantilever

Next, the muscle was subjected to an isometric contraction. The displacement of the cantilever, muscle and gel was simultaneously tracked in real‐time while the muscle was stimulated at 1 Hz. Muscle length was defined as the distance between the left side of the muscle (light blue point in Figure 2) and the cantilever on the right (green line in Figure 2). The change in muscle length was used as the process variable for the PID controller, while the set point of the PID controller was set to zero. Feedback control was enabled after a few twitches, and the amplified voltage output of the PID controller was applied to the motor directly during each control loop at a rate of 164 Hz. Gel displacements were converted into force estimates using the calibration. Stimulation data, real‐time displacements, force estimates and images were saved. The force produced by the muscle without feedback control and with feedback control was normalised to the force curve without feedback control and compared to determine the performance of the isometric protocol.

#### 2.11.6 Protocol 3: Isometric contraction between two points in tissue

We repeated this approach to achieve an isometric contraction between two points within the muscle, one at the left‐hand side (the same as in the previous isometric protocol) and the other at an arbitrarily chosen point in the muscle (orange point in Figure 2). Cantilever, muscle end tissue and gel displacements were all tracked in real‐time while the muscle was stimulated at 1 Hz. Gel displacement was converted into a force estimate using the calibration performed during fixed‐end contraction as previously. Force estimates were normalised to the traces without feedback control for comparison.

#### 2.11.7 Protocol 4: Isotonic contraction

Finally, isotonic contraction was tested by selecting a point in the gel (dark blue point in Figure 2) as the process variable for the PID controller with a displacement set point of zero, to mimic an isotonic contraction at zero force production. Before enabling control, calibration was performed by measuring gel displacement and estimating force from cantilever displacement. This allowed the gel to be used as a force sensor during feedback control. During the isotonic protocol, both the cantilever and gel were tracked in real‐time at 164 fps. Once tracking commenced, the muscle was electrically stimulated at 1 Hz, then feedback control was enabled after a few twitches and disabled again after a few twitches. Stimulation data, real‐time displacements, force estimates and images were recorded. The forces produced by the muscle without feedback control and with feedback control were compared to determine the effects of the isotonic protocol. Both muscle shortening and force were normalised to pre‐feedback control traces.

## 3 RESULTS

### 3.1 Protocol 1: Fixed‐end contraction

**FIGURE 3:**
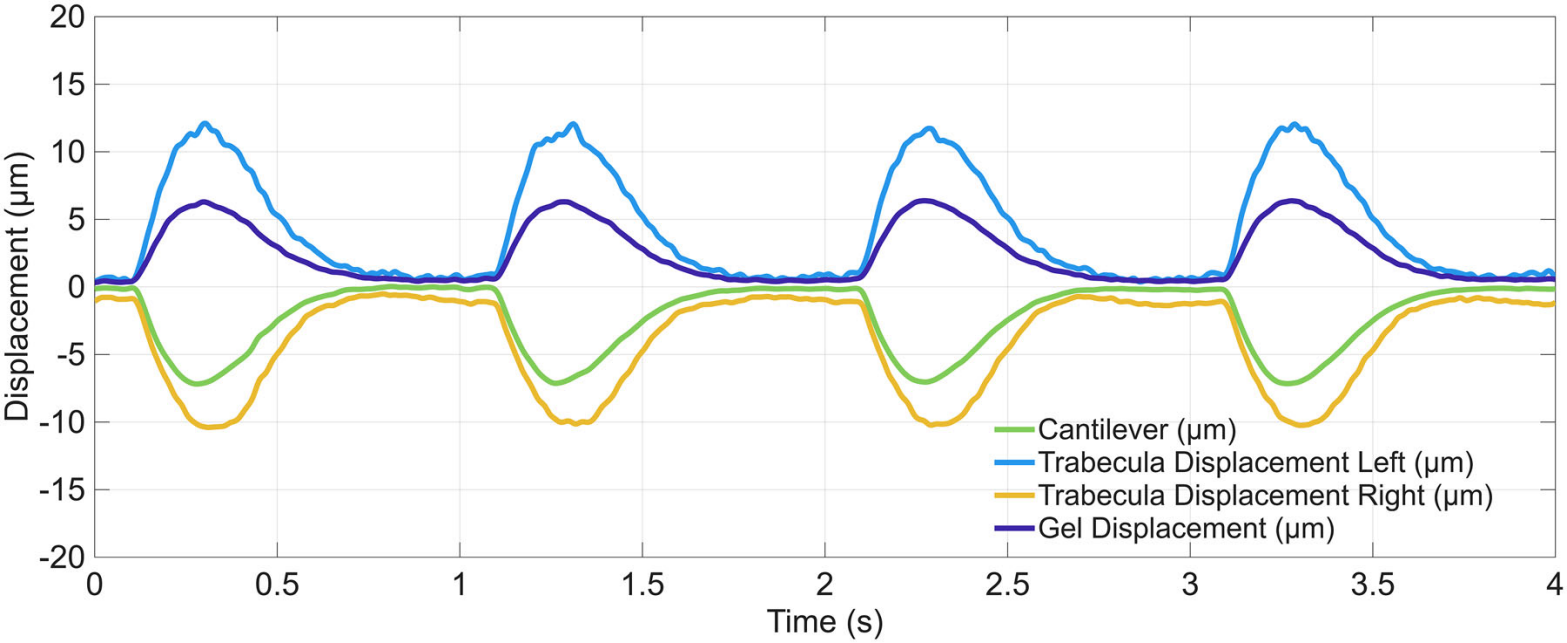
Real‐time displacement traces of the four regions noted in Figure 2 during fixed‐end contraction: cantilever (green), muscle LHS (blue), muscle RHS (orange) and gel (dark blue). Data from four contractions are displayed. The starting sarcomere length, at diastole, was approximately 2.2 µm.

The displacements of three selected points (tracked in 2D, in muscle tissue and gel) and the displacement of the cantilever (tracked in 1D) were measured successfully and robustly. For all figures, stimulation occurred at integer time points. Figure 2 shows images of the trabecula used in the experiments, with tracked areas and points indicated. The P‐SG‐GC algorithm computed the 2D displacement of the points and the 1D movement of the cantilever in 0.0055 ± 0.0002 s (mean ± SD, measured over a 10 s window), a period which was always less than the camera frame period of 0.0061 s (1/164 Hz). The corresponding displacement traces that were calculated in real‐time are shown in Figure 3 for the cantilever, muscle and gel. At peak contraction, (i.e., 0.3 s; first twitch) the trabecula shortened by around 19 µm from its original length of 1760 µm, a strain of 1.1 %, where the muscle length was measured between the cantilever and the trabecula endpoint.

### 3.2 Protocol 2: Isometric contraction protocol A (based on trabecula length defined between end tissue and cantilever)

**FIGURE 4:**
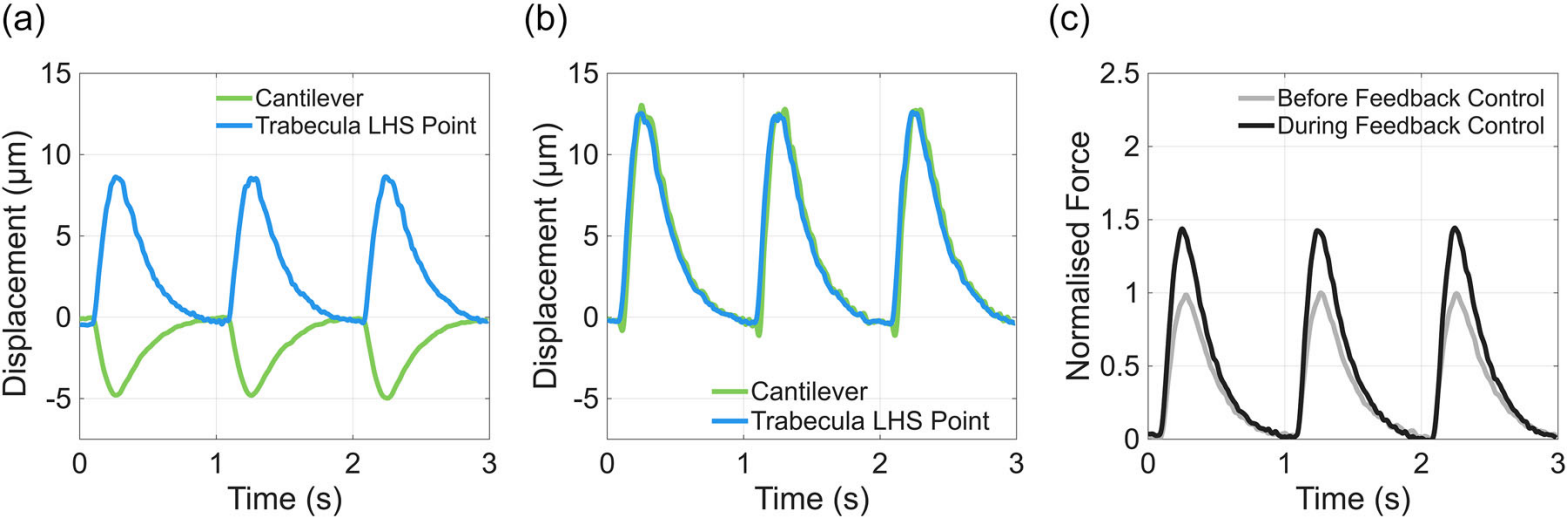
(a, b) Cantilever and trabecula left end displacement traces without (a) and with (b) feedback control of isometric contraction protocol 2. (c) Normalised force traces without and with feedback control. Feedback control was implemented to induce an isometric contraction based on muscle length (defined between the left end of the muscle and cantilever; see Figure 2). The starting sarcomere length, at diastole, was approximately 2.2 µm.

During isometric contraction (based on muscle length between the left light blue point and cantilever; Figure 2), the trabecula produced increased force compared to fixed‐end contraction (Figure 4). The left end of the trabecula moved to the right by 9 µm, while the cantilever moved to the left by 5 µm, without feedback control (Figure 4a). Note that this is identical to the fixed‐end contraction in protocol 1 (Figure 3). When feedback control was enabled, the movement of the cantilever closely followed the movement of the left end of the trabecula (Figure 4b). The fractional increase in force with feedback control was 1.4, corresponding to a force of 1.45 mN (Figure 4c).

### 3.3 Protocol 3: Isometric contraction protocol B (based on trabecula muscle length defined between two points in tissue)

**FIGURE 5:**
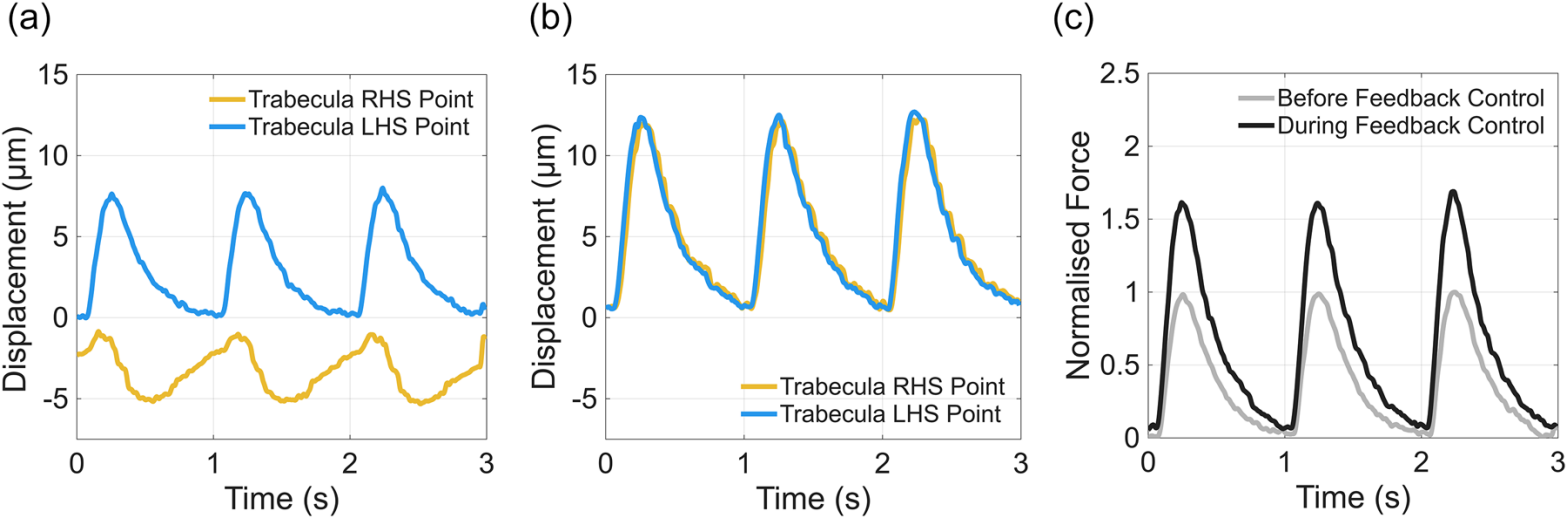
(a, b) Trabecula righthand side and lefthand side displacement traces without (a) and with (b) feedback control of isometric contraction protocol 3. (c) Normalised force traces without and with feedback control. Feedback control was implemented to induce an isometric contraction based on muscle length (defined between the left end of the muscle and right end of the muscle; see Figure 2). The starting sarcomere length, at diastole, was approximately 2.2 µm.

Isometric contraction (between left light blue point and right orange point; Figure 2) also resulted in an increase in force produced by the muscle. In the absence of feedback control, the right side of the trabecula moved to the left during contraction and the left side moved to the right. After feedback control was turned on, both ends moved to the right, with the displacement traces of right and left ends mimicking each other, similar to what was observed in Protocol 2 (Figure 4). Muscle force production increased by a factor of 1.6 during feedback control, as shown in Figure 5c.

### 3.4 Protocol 4: Isotonic contraction

**FIGURE 6:**
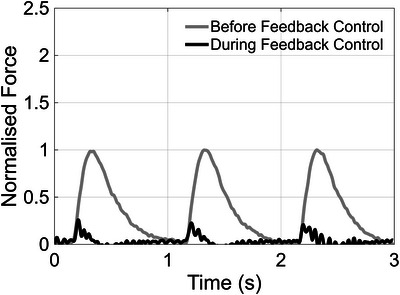
Normalised trabecula force production without and with feedback control.

Figure 6 shows the effect of using feedback control to perturb muscle length to maintain a zero‐force isotonic contraction. Force was determined by using the calibration performed during fixed‐end control and the tracked displacement of the hydrogel adhering to the left end of the muscle (Figure 2 dark blue point). The force diminished to near zero after force‐feedback control was turned on. The extent of shortening during isotonic contraction was approximately 50 µm.

## 4 DISCUSSION

Existing devices for trabecula experimentation are challenging to use as they require manually attaching the muscle at each end to sutures/hooks/clamps/pins, and usually involve the direct manipulation, using forceps, of the delicate tissues. This manipulation can often result in human‐induced tissue damage. Here, to overcome this limitation, a trabecula was successfully secured in place at one end using a photo‐curable hydrogel, while the other end was manipulated with a simple cantilever force sensor. The linear stages allowed easy manoeuvring of the cantilever for attachment.

The device allows a muscle to be stretched to any user‐defined sarcomere length, fixed in place, sustained, stimulated to contract, and imaged. We use our robust and accurate image registration algorithm (Cheuk et al., 2020; Lam Po Tang et al., 2019) to track muscle deformation to derive muscle length, strain and force production in real‐time. We track selected points in the imaged muscle in real‐time, to approximately 20 nm resolution using only the intrinsic visible features of the muscle. By tracking and feedback‐controlling the attachment mechanisms, we robustly measure and control muscle force and length.

### 4.1 Hydrogel

In this study, we used the Ru/SPS visible‐light photo‐initiating system to photo‐cure gel into user‐defined patterns around the end of the trabecula to hold it in place throughout an experiment. The hydrogel provides an easy and efficient mounting process, with the potential to mount multiple muscles in multiple tissue chambers for high throughput testing purposes. The use of a photo‐crosslinked gel provides spatio‐temporal stiffness control in a relatively short timeframe – an advantage over other systems that crosslink using chemicals or enzymes. Seeding the gel with particles allowed force to be estimated from gel displacement. The mechanical properties of the gel were linear in the strain range of interest, allowing a simple material model to be fitted. The gel displacements were calibrated against the force production obtained by measuring cantilever movement during muscle contraction (Lam Po Tang et al., 2023).

### 4.2 Image‐based measurements

In this device, we track the displacements of the sample, cantilever and gel to sub‐pixel (∼20 nm) resolution from real‐time images gathered at 164 frames per second. The advantage of our image registration‐based measurement is that we measure displacement at multiple areas of interest in any camera image, at a much lower cost, but at higher resolution, than other methods. In this configuration the measurement rate of 164 Hz is sufficient for performing real‐time measurement and control of muscle contraction.

### 4.3 Length and force control

We have presented here the ability to perform feedback control of muscle length or force based on real‐time tracked displacements, using the P‐SG‐GC image registration algorithm. In other systems, trabecula length is sometimes measured by interferometers tracking the movement of hooks that are attached to the muscle (Han et al., 2019; Pham et al., 2019), but such systems are expensive, and complicated to set up. The method used here allows several displacements to be simultaneously measured, using only a USB3 camera and computer software. The motor used to control muscle length also is relatively inexpensive; here we use a piezoelectric motor, but a small voice‐coil motor would be equally suitable.

In the isometric protocols, feedback control was used to compensate for muscle shortening by moving the motor based on tracked displacements. An isometric contraction resulted in 40% greater force produced by the muscle compared with fixed‐end contraction, consistent with previous findings (Tabern et al., 2011). Force increased by around 40% during isometric protocol 2, where muscle length was measured between the left end of the trabecula and the cantilever. Alternatively, muscle length can be measured between two points within the muscle, and changes in muscle length in this region can be minimised. Some preparations can exhibit non‐uniform contraction in certain regions (Cheuk et al., 2020; ter Keurs et al., 1980); using the isometric protocol described here, these regions may be excluded during control and analysis.

During the isotonic protocol the change in force was minimised, with a peak of disturbance of 25% of the force without feedback control. During isotonic control, the shortening of the muscle was approximately four‐fold higher than under fixed‐end contraction.

### 4.4 Limitations and future work

The acquisition and feedback control loop rate is currently limited by the camera and USB interface to 164 Hz. If higher control bandwidth is required, a reduced frame size with fewer pixels would allow faster frame rates. The other factor limiting loop rate is the time required to compute material point deformation. At present we can track up to four material points in real‐time; higher loop rates or additional material points would require further code parallelisation or reduced sub‐image size. Finally, while our system can in principle sustain up to eight fixed‐end trabeculae at once, our current imaging system and motor system allow measurements from one well at a time. Truly parallelised experiments on dynamically loaded trabeculae would require a motor and force sensor on each of the eight wells. Such a system is currently under development.

All data presented in this paper were collected at room temperature. Although temperature may impact the cross‐linking kinetics of the hydrogel, the gel stiffness is measured post‐curing to account for any changes in hydrogel properties. An incubator has been built to sustain the samples and microscope at body temperature and will be explored and reported on in future work.

While real‐time measurement and control systems have been added to the device, the full capabilities of the device have yet to be explored. We propose three applications. First, multiple muscles could be studied by fixing both ends of each trabecula to the glass with hydrogel, allowing multiple muscles to be cultured and studied sequentially during fixed end contractions. Currently available devices allow only one muscle to be studied at a time, limiting throughput in vitro (Choi et al., 2020; de Tombe & Stienen, 2007; Mellor, 2020; Pham et al., 2017). Second, there is also the possibility of inferring force solely from the gel deformation, and the known dimensions and properties of the cured gel, without the need for in situ gel calibration. This approach could be extended to totally encasing the preparation in hydrogel of known stiffness, allowing examination of the effect of the body load provided by the gel on muscle contraction. Lastly, further development will be undertaken to add various contraction modes such as force clamps, autotonic shortening protocols and work‐loop protocols that mimic the pressure–volume cycle of the heart (Dowrick et al., 2022; Garrett et al., 2019).

### 4.5 Conclusions

We designed and constructed a device for experimenting with cardiac trabeculae secured by light‐curable hydrogel and a piezoelectrically actuated force sensor. Trabeculae can be mounted and measurements can be made with little direct contact with the muscle, reducing the possibility of human‐induced tissue damage. With this device, we measure and control muscle displacement and force in real‐time using non‐contact image registration techniques and software‐based motor control.

## AUTHOR CONTRIBUTIONS

The authors confirm their contribution to the paper as follows: study conception and design: all authors; data collection: E.J. Clark Murphy, T. Pham, J‐C. Han; analysis and interpretation of results: E.J. Clark Murphy, T. Pham, K. Tran, J‐C. Han, A.J. Taberner; draft manuscript preparation: E.J. Clark Murphy, T. Pham, A. J. Taberner. All authors have read and approved the final version of this manuscript and agree to be accountable for all aspects of the work in ensuring that questions related to the accuracy or integrity of any part of the work are appropriately investigated and resolved. All persons designated as authors qualify for authorship, and all those who qualify for authorship are listed.

## CONFLICT OF INTEREST

None declared.
